# Prostate Specific Antigen (PSA) as Predicting Marker for Clinical Outcome and Evaluation of Early Toxicity Rate after High-Dose Rate Brachytherapy (HDR-BT) in Combination with Additional External Beam Radiation Therapy (EBRT) for High Risk Prostate Cancer

**DOI:** 10.3390/ijms17111879

**Published:** 2016-11-10

**Authors:** Thorsten H. Ecke, Hui-Juan Huang-Tiel, Klaus Golka, Silvia Selinski, Berit Christine Geis, Stephan Koswig, Katrin Bathe, Steffen Hallmann, Holger Gerullis

**Affiliations:** 1 Department of Urology, HELIOS Hospital, D-15526 Bad Saarow, Germany; steffen.hallmann@helios-kliniken.de; 2 Department of Neurology/Emergency Unit, Vivantes Hospital Spandau, D-13585 Berlin, Germany; h.huang-tiel@t-online.de; 3 Leibniz Research Centre for Working Environment and Human Factors IfADo, D-44139 Dortmund, Germany; golka@ifado.de (K.G.); selinski@ifado.de (S.S.); berit.geis@tu-dortmund.de (B.C.G.); 4 Department of Radio-Oncology, HELIOS Hospital, D-15525 Bad Saarow, Germany; stephan.koswig@helios-kliniken.de (S.K.); katrin.bathe@helios-kliniken.de (K.B.); 5 School of Medicine and Health Sciences Carl von Ossietzky, University Oldenburg, D-26133 Oldenburg, Germany; holger.gerullis@gmx.net

**Keywords:** PSA, toxicity, HDR brachytherapy, prostate cancer

## Abstract

High-dose-rate brachytherapy (HDR-BT) with external beam radiation therapy (EBRT) is a common treatment option for locally advanced prostate cancer (PCa). Seventy-nine male patients (median age 71 years, range 50 to 79) with high-risk PCa underwent HDR-BT following EBRT between December 2009 and January 2016 with a median follow-up of 21 months. HDR-BT was administered in two treatment sessions (one week interval) with 9 Gy per fraction using a planning system and the Ir192 treatment unit GammaMed Plus iX. EBRT was performed with CT-based 3D-conformal treatment planning with a total dose administration of 50.4 Gy with 1.8 Gy per fraction and five fractions per week. Follow-up for all patients was organized one, three, and five years after radiation therapy to evaluate early and late toxicity side effects, metastases, local recurrence, and prostate-specific antigen (PSA) value measured in ng/mL. The evaluated data included age, PSA at time of diagnosis, PSA density, BMI (body mass index), Gleason score, D’Amico risk classification for PCa, digital rectal examination (DRE), PSA value after one/three/five year(s) follow-up (FU), time of follow-up, TNM classification, prostate volume, and early toxicity rates. Early toxicity rates were 8.86% for gastrointestinal, and 6.33% for genitourinary side effects. Of all treated patients, 84.81% had no side effects. All reported complications in early toxicity were grade 1. PSA density at time of diagnosis (*p* = 0.009), PSA on date of first HDR-BT (*p* = 0.033), and PSA on date of first follow-up after one year (*p* = 0.025) have statistical significance on a higher risk to get a local recurrence during follow-up. HDR-BT in combination with additional EBRT in the presented design for high-risk PCa results in high biochemical control rates with minimal side-effects. PSA is a negative predictive biomarker for local recurrence during follow-up. A longer follow-up is needed to assess long-term outcome and toxicities.

## 1. Introduction

High-dose-rate brachytherapy (HDR-BT) with additional external-beam radiation therapy (EBRT) is an important therapeutic option for men diagnosed with clinically localized and locally advanced high-risk prostate cancer (PCa) [1,2,3].

Regarding the actual European guidelines for the treatment of patients with intermediate- and high-risk PCa, a life expectancy of at least 10 years should be mandatory for treatments like radical prostatectomy (RP) or radiation therapy. Nevertheless, until now, there are no randomized clinical trials that compare the oncological outcome of HDR-BT vs. RP [4]. In general, there are not much data available regarding the oncological outcome of HDR-BT [5,6]. Especially, HDR-BT in combination with EBRT seems better than EBRT alone, with respect to biochemical recurrence (BCR)-free survival rates and aspects of quality of life [5,7]. PCa cells seem to have a low α/β ratio. This encourages the use of HDR-BT, where higher doses per fraction can be performed, therefore making it one of the most efficient interventions of hypofractionated radiotherapy. Most reported series combining HDR-BT and EBRT describe impressive results for the treatment of intermediate and high-risk PCa [8,9,10].

It has been reported by Schiffmann et al. that additional androgen deprivation therapy (ADT) shows higher BCR-free survival rates [11]. In this study, we focused on patients with intermediate- and high-risk PCa that were treated with HDR-BT plus ERBT plus ADT regarding complication rates and oncological outcome. It is difficult to determine an early treatment failure after therapy based on prostate-specific antigen (PSA) fluctuation and a potential benign PSA-rebound phenomenon [12]. In other studies, a benign PSA-rebound rate of up to 30% was described within the first 36 months after treatment [12,13,14]. The aim of this study was to determine early toxicity rates and the influence of PSA as a predictive marker of clinical outcome.

## 2. Results

The parameters age, IPSS, PSA (ng/mL) at time of diagnosis, PSA density, BMI, Gleason score, D’Amico risk classification for PCa, PSA value after one year FU, and time of FU are shown in Table 1. In that table, for all main clinical parameters, minimum, median, mean, maximum, standard deviation (SD), and 10%, 25%, 75%, and 90% intervals have been calculated. The median follow up time in our study was 21 months (6–80 months). In total, 8 out of 79 patients (10%) reached a FU time of more than five years. The frequencies of all important clinical parameters—PSA at time of diagnosis, Gleason score, T staging, and D’Amico risk classification for PCa—are detailed in Table 2, Table 3, Table 4 and Table 5. Of the study cohort, 64.5% had an initial PSA value of more than 10 ng/mL, the Gleason score of more than 90% of the patients was ≥7, more than 80% of the patients had a clinical T staging of 3 (positive digital rectal examination and/or positive for tumor in transrectal ultrasound examination). According to the D’Amico risk classification for PCa, more than 90% are classified to risk group 3. In conclusion, all patients in that study for HDR-Brachytherapy treatment are high-risk PCa patients.

During follow-up, one patient (1.27%) died due to progressive disease and bone metastases 63 months after initial diagnosis of PCa. This patient was also the only one with the detection of metastases. In total, a local recurrence was detectable in three patients (3.80%).

Another focus of that study report is the description of side effects regarding early toxicity rates of the demonstrated treatment. Of all treated patients, 84.81% had no side effects. All reported complications in early toxicity were grade 1. The most reported side effects were anal pain (5.06%), symptomatic proctitis (1.27%), and diarrhea (2.53%) for the gastro-intestinal tract; high urinary frequency (3.80%), and urgency (2.53%) were the most complained side effects for the urinary tract. All complications are shown in Table 6.

After descriptive analyses of the documented data, we focused on the influence of PSA value while follow-up for local recurrence, metastases, and/or death. As only one patient died during follow-up, no statistical significance was calculable. However, the presence of local recurrence (*n* = 3) was used for the evaluation of PSA for risk assessment. Table 7 shows the *p*-value of all important parameters during follow-up regarding the influence on the presence of local recurrence. We could show that PSA density at time of diagnosis (*p* = 0.009), PSA on date of first HDR-BT (*p* = 0.033), and PSA on date of first follow-up after one year (*p* = 0.025) have statistical significance on a higher risk of having a local recurrence during follow-up. We found no statistical significance for Gleason score (*p* = 0.463) or D’Amico risk classification (*p* = 0.995).

## 3. Discussion

HDR brachytherapy is one of the minimally invasive techniques of delivering conformal hypofractionated radiotherapy with steep fall-off of dose beyond the prostate gland. The prostate gland lays very close to critical normal tissues—the anterior rectum wall, urethra, and bladder neck. Because of that biological fact, HDR-BT is ideal for the treatment of PCa [15]. Many groups have shown that HDR-BT boost in combination with EBRT provides better results compared to EBRT alone [2,3]. Moreover, brachytherapy boost has the convenience of decreasing total treatment time, leading to decreased traveling time and expenses.

In the published data describing the experience of HDR-BT boost in combination with EBRT, various fractionation schedules have been used: 15 Gy in three fractions, 11–22 Gy in two fractions, and 12–15 Gy in one fraction. All of them had excellent results, so the Groupe Européen de Curiethérapie—European Society Therapy Radiation Oncology (GECESTRO) and the American Brachytherapy Society (ABS) do not recommend one fractionation schedule over another [16,17,18]. In this study, all patients were treated with two fractions of 9 Gy each following EBRT as reported above. In our cohort, 84.8% of the treated patients had no side effects. All reported side effects were defined as acute toxicity grade 1; most relevant were pain (5.06%), proctitis (1.27%), and diarrhea (2.53%) as intestinal; and frequency (3.8%) and urgency (2.53%) as genitourinary side effects. None of the patients developed acute GU or GI morbidity higher than Grade 2.

Hoskin et al. [19] reported about early ≥Grade 3 GU and GI morbidity was 3%–7% and 0%, respectively. Late Grade 3 GU toxicity was 3%–16% with no late Grade 3 or 4 GU or GI toxicity. Barkati et al. [20] reported 88% and 85% three-year and five-year biochemical control rates, respectively. They reported all acute GU toxicity as Grade 1. Chronic Grade 3 urinary toxicity was <10% with no Grade 4 toxicity seen.

## 4. Materials and Methods

### 4.1. Subjects

In this retrospective study, we report on 79 male patients (median age 71 years, range 50 to 79) who were treated between December 2009 and January 2016 at the Department of Urology and the Department of Radio-Oncology of HELIOS Hospital Bad Saarow, Germany. All patients selected for that treatment have been classified as intermediate and high-risk PCa patients.

### 4.2. Study Design

All included patients (*n* = 79) underwent HDR-BT after informed patient consent at the time of their treatment. Digital rectal examination, PSA, computerized tomography (CT), and a Technecium-99 bone scan was mandatory. Risk stratification was done as per the National Comprehensive Cancer Network (NCCN), which defines low-risk as PSA ≤ 10 ng/mL, T1c-T2 and a Gleason score (GS) ≤ 6; intermediate risk as PSA 10–20 ng/mL or GS 7; and high risk as a PSA > 20 ng/mL, T3, or GS 8–10. PSA measurements were performed with ElektroChemiLumineszenzImmonoAssay (ELCIA) by Roche Diagnostics GmbH, in accordance with WHO standards. We evaluated and documented the D’Amico risk stratification for PCa for each patient. Exclusion criteria were surgically positive lymph node metastases, distant metastasis, and prior pelvic radiotherapy. Patients with bladder outlet obstruction, patients who already had transurethral operations, and patients with a prostate volume of more than 100 cm^3^ were also excluded. All patients had neoadjuvant and adjuvant androgen deprivation therapy (ADT) for at least two years starting after laparoscopic pelvic lymphadenectomy.

HDR-BT was administered before EBRT, based on transrectal ultrasound imaging, using a planning system and the Ir192 treatment unit GammaMed Plus iX (by Varian). HDR-BT was administered in two treatment sessions (one week interval) with 9 Gy per fraction. Overall, 18 Gy was applied to the prostate plus 2 mm margin. The maximal dose for the urethra and rectal wall was 8.0 and 5.0 Gy, respectively. 

The HDR-BT procedure was done under general anesthesia. The patient was placed in the lithotomy position, a square lightweight template having a 5 mm grid array was fixed on a stepper stand on which a transrectal ultrasound machine (TRUS) was mounted, and the template was jammed against the perineal skin. There was a grid faceplate fixed onto the template, corresponding to the grid of the TRUS for accurate placement of the ProGuide needles. Under TRUS guidance, metallic trocars were inserted transperineally through the holes in the template to ascertain the position in the prostate as published before by Deger et al. [21]. Seven to twenty needles were inserted into the prostate, then the trocars were removed and replaced by the 6F ProGuide plastic needles in the same position. We always started with the peripheral and anterior needles, and then moved towards the center. As far as possible, the needles were placed at 1 cm intervals. No needles were placed within 7 mm of the urethra, in order to have control over the urethral dose. The needles were pushed beyond the prostate base, and the posterior needles were placed 2–3 mm anterior to the anterior wall of the rectum to avoid overdosing the rectum. 

**Figure 1:**
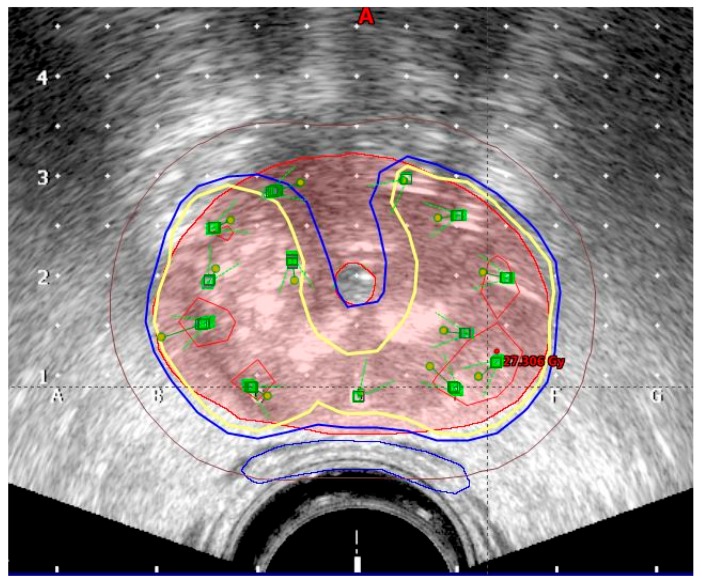
Isodoses in transrectal ultrasound image (red: 15 Gy; yellow: 9 Gy; blue: 8 Gy; brown: 5 Gy).

**Figure 2:**
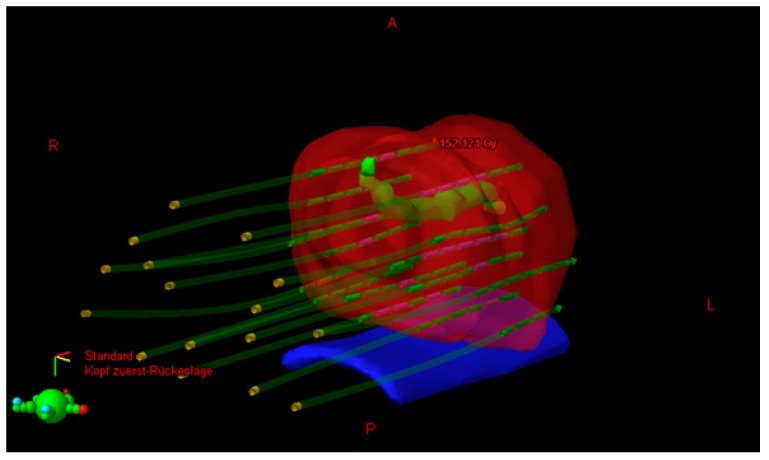
Three-dimensional image with simulation of radiation (red: prostate, green: urethra, blue: rectum, dark green: needle positions).

The planning target volume (PTV) was contoured by the radiation oncologist on each ultrasound slice and included the prostate with a 3 mm margin all around, except posteriorly, where no margin was given to avoid overdosing the anterior rectal wall. Superiorly, a margin of 5–7 mm was given to compensate for any post-implant edema and inadvertent caudal movement of the catheters in between the fractions. The PTV constraints were D90 (dose delivered to 90% of PTV) ≥ 97%, V95 ≥ 100%, and V150 ≤ 35%. Isodoses in transrectal ultrasound image are shown in Figure 1. A three-dimensional image with simulation of radiation is shown in Figure 2.

EBRT started a week after HDR-BT. EBRT was performed according to the standardized protocol with CT-based 3D-conformal treatment planning. The clinical target volume included prostate, the periprostatic region, and the basis of seminal vesicles; the planning target volume included the CTV (clinical target volume) and a margin—margin of 0.6 cm (posterior) and 1.0 cm in all other directions. The reference dose is defined in accordance with the International Commission on Radiation Units and Measurements report 50/63. All patients were irradiated in a supine position with a CT-planed IMRT/VMAT-technique (intensitivity modulated radiotherapy/volume modulated arc therapy) with 6 MV megavoltage photons (Varian, CClinac DHX, PaloAlto, CA, USA). A total dose of 50.4 Gy with 1.8 Gy per fraction and five fractions per week was administered.

Follow-up for all patients was organized one, three, and five years after radiation therapy in the department of Radiooncology to evaluate early and late toxicity side effects, metastases, local recurrence, and PSA value.

### 4.3. Evaluated Data

The evaluated data included the parameters age, PSA (ng/mL) at time of diagnosis, PSA density, body mass index (BMI), Gleason score, D’Amico risk classification for PCa, digital rectal examination (DRE), PSA value after one/three/five year(s) follow-up (FU), time of follow up, TNM classification, prostate volume, and early toxicity in follow-up. Pretreatment international prostate symptom score (IPSS), uroflow, and rest urine after voiding were also documented. All relevant dates were documented: date of birth, date of death, date of diagnosis, date of lymphadenectomy, date of ADT, date of HDR-BT, and date of follow-up after one, three, and five years.

A radiation oncologist and a urologist performed the follow-up evaluations, including digital rectal examinations and PSA level during follow-up scheme one, three, and five years after initial treatment. PSA failure was defined in terms of the American Society for Therapeutic Radiology and Oncology Consensus Panel recommendations [22]. Acute toxicities were scored according to the Common Terminology Criteria for Adverse Events, Version 4.0 (CTCAE v4.3), by the National Cancer Institute (Common Terminology Criteria for Adverse Events, Version 4.0. Available online: http://evs.nci.nih.gov/ftp1/CTCAE/CTCAE_4.03_2010-06-14_QuickReference_8.5x11.pdf). Acute toxicity was defined as symptoms that were observed during or after treatment and had been completely resolved 6 months after treatment. Following a strict plan for FU, all treated patients were investigated after six months, one, three and five years. Besides clinical investigation including DRE, an interview with a focus on acute toxicities following the Common Terminology Criteria for Adverse Events as written above was included.

### 4.4. Statistical Analysis

Bravais–Pearson correlation coefficients were estimated for pairs of variables. Odds ratios (OR), 95% confidence intervals (95% CI), and *p*-values of the Wald test were estimated using unadjusted logistic regression for local recurrence as dependent variable. 

The level of significance was α = 0.05. All tests and calculations were performed using the software R, version 3.1.2 (R Development Core Team 2014).

In our group, we could show that PSA density at time of diagnosis (*p* = 0.009), PSA on date of first HDR-BT (*p* = 0.033), and PSA on date of first follow-up after one year (*p* = 0.025) have statistical significance with respect to a higher risk of having a local recurrence during follow-up, but not age, Gleason score, or clinical stage. Concerning the number of patients (*n* = 79 in total), we are in average position compared to others and in a good position for a single-center study. Though the series of Yoshiaka et al. performed a monotherapeutic HDR-BT, they found the initial PSA level to be a significant prognostic factor (*p* = 0.029) along with younger age (*p* = 0.019) [23].

In the data published by Hoskin et al., Zwahlen et al., and Kestin et al. [3,5,8,24], they could show a better biological recurrence-free survival after HDR-BT in combination with EBRT, compared to EBRT alone. Combination of both modalities may also improve overall survival (OS) [25]. An explanation could be the high radiation dose that can be prescribed when HDR-BT is combined with EBRT.

Deger et al. [26] presented data of 422 patients with localized PCa treated between 1992 and 2001 with HDR-BT and 3DRT. As also performed in our treatment protocol, all patients underwent laparoscopic pelvic lymph node dissection to have an exact pathological lymph node staging and to be sure to exclude patients with lymphatic involvement. The biological non-evidence of disease (bNED) according to risk group were 100% for low risk, 75% for intermediate risk, and 60% for high risk at 5 years. Five-year bNEDs were 81% in the low risk, 65% in the intermediate risk, and 59% in the high risk group. Five-year OS and bNED were 87% and 94%, respectively. The authors also observed that initial PSA value, risk group, and age were significantly related to bNED. In contrast to our results, we found no statistical significance for D’Amico risk classification (*p* = 0.995) or Gleason score (*p* = 0.463). This could be caused by the fact that the presented cohort consists mainly of patients with high risk PCa and also high Gleason scores.

Most studies of radiotherapy in PCa focus on two points: not only the effectiveness of the treatment, but also its tolerance. However, due to different classifications of radiation reactions, it seems to be difficult to compare the toxicity rates.

There are still different opinions about the use of ADT for patients with intermediate- and high-risk PCa. Martinez et al. [27] published with a large number of patients (*n =* 1260) treated with pelvic RT and HDR-BT. The first group was treated with additional ADT up to six months prior to radiation, and the second group was not. The results for OS, disease free survival (DFS), and bNED have been similar. They observed that additional ADT did not confer a therapeutic advantage, but only side effects and cost. No statistically significant benefit on bNED rates with the use of additional ADT could be shown in any of the groups in that study. 

The main limitation of this study is the relatively small number of patients and the short follow-up time regarding the influence on cancer-specific survival, overall survival, and biochemical relapse. Our study adds to the already existing evidence for the effectiveness of HDR-BT combined with EBRT for high-risk PCa.

In conclusion, this study demonstrates that HDR-BT combined with EBRT is effective in the radical radiotherapy of intermediate- and high-risk localized and locally advanced PCa. A longer follow-up is needed to assess long-term outcome and toxicities.

## 5. Conclusions 

HDR-BT in combination with additional EBRT in the presented design for local advanced and high-risk PCa results in high biochemical control rates with minimal side-effects. PSA is a negative predictive biomarker for local recurrence during follow-up.

## Figures and Tables

**Table 1:** Main clinical parameters. BMI: body mass index; FU: follow-up; IPSS: international prostate symptom score; PSA: prostate-specific antigen.

Parameter	Min	10%	25%	Median	Mean	75%	90%	Max	SD
**Age**	50.000	59.800	66.000	71.000	69.241	74.000	76.000	79.000	6.622
**IPSS**	0.000	2.000	3.000	5.500	6.271	9.000	11.100	19.000	4.426
**PSA Diagnosis**	1.360	4.464	7.045	14.550	22.345	24.995	44.736	226.000	29.506
**PSA Density**	0.053	0.126	0.250	0.463	0.764	0.828	1.760	4.969	0.924
**BMI**	20.761	23.397	25.282	27.099	27.385	28.572	31.760	44.379	3.713
**Gleason Score**	6.000	7.000	7.000	7.000	7.354	8.000	9.000	9.000	0.848
**D’Amico**	2.000	3.000	3.000	3.000	2.962	3.000	3.000	3.000	0.192
**PSA FU 1a**	0.000	0.010	0.030	0.040	0.167	0.165	0.304	2.300	0.357
**time FU**	6.000	8.800	11.000	21.000	26.620	35.500	57.400	80.000	18.912

**Table 2:** Frequency of important clinical parameters for the study cohort. Pre-treatment PSA value.

PSA Diagnosis	*N*	%
PSA < 10	28	35.44
10 ≤ PSA < 20	22	27.85
PSA ≥ 20	29	36.71
Total	79	100.00

**Table 3:** Frequency of important clinical parameters for the study cohort. Gleason Score.

Gleason Score	*N*	%
6	7	8.86
7	49	62.03
8	11	13.92
9	12	15.19
Total	79	100.00

**Table 4:** Frequency of important clinical parameters for the study cohort. Clinical T Stage.

T Stage	*N*	%
2a	1	1.27
2b	5	6.33
2c	7	8.86
3	66	83.54
Total	79	100.00

**Table 5:** Frequency of important clinical parameters for the study cohort. D’Amico risk classification for PCa.

D’Amico	*N*	%
1	0	0
2	3	3.80
3	76	96.20
Total	79	100.00

**Table 6:** Early toxicity rates after radiation therapy.

Side Effects	*N*	%
None	67	84.81
Intestinal		
Pain	4	5.06
Proctitis	1	1.27
Diarrhea	2	2.53
Hemorhage	0	0
Genitourinary		
Frequency	3	3.80
Urgency	2	2.53
Incontinence	0	0
Hematuria	0	0
Renetntion	0	0
Pain	0	0
Total	79	100.00

**Table 7:** *p*-Value for relevant parameters regarding local recurrence during follow-up.

Variable	OR	2.5%	97.5%	*p*-Value
BMI	1.035	0.778	1.376	0.814
Age	0.876	0.755	1.016	0.080
IPSS	0.401	0.145	1.104	0.077
PSA pre-therapeutic	1.011	0.990	1.033	0.311
PSA density	3.102	1.331	7.228	*0.009*
No. of lymphnodes	0.973	0.795	1.190	0.789
PSA Lymphadenectomy	1.004	0.997	1.032	0.758
PSA HDR1	1.123	1.009	1.250	*0.033*
PSA HDR2	1.095	0.985	1.217	0.093
PSA FU 1a	8.022	1.306	49.287	*0.025*
PSA FU 3a	1.977	0.258	15.134	0.511
PSA FU 5a	4.204	0.422	41.914	0.221

HDR: high dose rate. Statistical significance is written in *italics*.
